# Protective Effects and Potential Mechanisms of Dietary Resveratrol Supplementation on the Spleen of Broilers Under Heat Stress

**DOI:** 10.3389/fnut.2022.821272

**Published:** 2022-05-16

**Authors:** Tiantian Meng, Juying Deng, Dingfu Xiao, Muhammed Adebayo Arowolo, Chunming Liu, Liang Chen, Wei Deng, Shaoping He, Jianhua He

**Affiliations:** ^1^College of Animal Science and Technology, Hunan Agricultural University, Changsha, China; ^2^Institute of Animal Husbandry and Aquaculture, Huaihua Academy of Agricultural Sciences, Huaihua, China; ^3^College of Animal Science and Technology, China Agricultural University, Beijing, China

**Keywords:** resveratrol, heat stress, anti-inflammatory, apoptosis, broiler

## Abstract

Resveratrol (RSV) is a natural polyphenolic compound with potent antioxidant and anti-inflammatory properties. This study aimed to investigate the protective effects of RSV supplementation on the inflammatory responses of broilers during heat stress. A total of 432 28-d-old white-feathered broilers (817 crossbred chicken) with an average weight of 549 ± 4 g were randomly allotted to 4 equal groups (Half male and half female, 6 replicates/group, 18 chickens/replicate), including normal temperature (NT) group (24 ± 2°C for 24 h/d, basal diet), NT+RSV group (24 ± 2°C for 24 h/d, basal diet + RSV), heat stress (HT) group (37 ± 2°C for 8 h/d, basal diet), and HT+RSV group (37 ± 2°C for 8 h/d, basal diet + RSV). Serum samples were collected on d 7 and 14 of heat stress, and thymus, spleen, jejunum, and bursa of Fabricius samples were collected and analyzed on d14. RSV treatment decreased the feed conversion ratio, partially reversed the negative alternations in body weight, average daily gain, and average daily feed intake caused by heat stress. RSV treatment also decreased the elevated levels of corticosterone on d 14, adrenocorticotropic hormone, and triiodothyronine in serum on d 7 caused by heat stress, and significantly increased the villus height to crypt depth ratio in the jejunum on d 14. Dietary RSV also reduced heat stress-induced splenic pro-inflammatory cytokine concentrations. TUNEL assay showed that RSV significantly reduced heat stress-induced the number of apoptotic cells. Remarkably, RSV down-regulated some splenic related genes for apoptosis genes, including *BCL-2, Apaf-1*, and *MDM2* mRNA levels induced by heat stress. According to GO and KEGG enrichment analyses, the differential genes between HT and HT + RSV groups were mainly associated with immune system process, hematopoietic or lymphoid organ development, and toll-like receptor signaling pathway. The relative mRNA expression of *NF-*κ*B*, heat shock protein 70 (*HSP70*), and *p38 MAPK* were markedly decreased by the combination of RSV and heat stress. These findings showed that RSV might reduce the splenic inflammatory response in heat-stressed white-feather broilers by inhibiting heat stress-induced activation of *NF-B, MAPK*, and *HSP70*, as well as inhibiting the activation of mitochondrial apoptotic pathways.

## Introduction

Heat stress has detrimental impacts on the poultry industry particularly in tropical subtropical and arid regions (1). Broilers are one of the most heat-sensitive animals due to their special physiology (2–4). Exposure to high ambient temperature has a detrimental impact on biological defense mechanisms in birds, such as the immunological response, which is linked to spleen atrophy, leading to immune response dysfunction (5, 6).

As a phytoalexin polyphenolic compound (7), resveratrol (RSV) (3,4,5-trihy-droxystilbene) exhibits multiple bioactivities, including anti-oxidative (8), anti-inflammatory (9, 10), and anti-aging (11) properties in animals. Our previous study has reported that RSV supplementation in heat stress-induced black-boned chickens improved growth performance and reduced oxidative stress by modulating the expression of heat shock genes in immune organs (12). RSV also alleviated heat-stressed impairment of the intestine by improving the barrier function (13).

Although RSV immunomodulatory actions are potentiated via nuclear factor kappa-B (NF-κB), mitogen-activated protein kinase (MAPK), and PI3K/AKT signaling pathways (6), a transcriptomic search for further molecular targets in the spleen of broilers remains to be fully elucidated. Therefore, this study proposed to evaluate the possible beneficial effects and potential mechanisms of RSV on growth performance, serum hormone concentration, the levels of splenic cytokines and apoptosis, splenic transcriptional response, and the immune signal pathway genes of heat-stressed white-feathered broilers.

## Materials and Methods

### Animal Ethics

All experimental and sample collection procedures were carried out according to the Chinese guidelines for animal welfare and approved by the Institutional Animal Care and Use Committee of the Hunan Agricultural University (permit number: CACAHU 2020-0821).

### Birds, Diets, and Management

A total of 500 healthy 1-day-old white feather broilers (817 crossbred chicken) were used before this experiment. From d 1 to 21 of age, except for light, broilers were managed with commercial standard feeding and raising managements. The chickens were kept in a room with 24-h of light from d 1 to 3 of age, 22-h of light and 2-h darkness from d 4 to 5 of age, 20-h of light and 4-h darkness from d 6 to 7 of age, 18-h of light and 6-h darkness from d 8 to 9 of age, and 16-h of light (06:30–22:30 h) and 8-h darkness from d 10 to 42 of age. During the week of acclimatization (d 21–27), birds were supplied a basal diet and water. Afterward, 432 28-d-old white feather broilers (817 crossbred chicken, half male and half female) with an average weight of 549 ± 4 g (individually weighed) were selected and randomly divided into 4 groups (6 replicates/group, 18 chickens/replicate). Nine birds were housed in a wire cage with 3 ladders (70 × 70 × 100 cm), 2 wire cages formed an experimental unit, randomly distributed in the shed. All broilers were allowed free access to water and feed (crumbled) throughout the experimental period.

The normal temperature (NT) and NT+RSV groups were housed in an environmentally controlled chamber at 24 ± 2°C for 24 h/d and fed with the basal diet and basal diet with 500 mg/kg of RSV (NT + RSV), respectively. In the remaining 2 groups, broilers were subjected to cyclic heat stress in the control room at 37 ± 2°C for 8 h/d (09:00–17:00 h) and followed by 24 ± 2°C for 16 h/d. Air conditioning and electric heat lamps were used to raise the temperature, which raised to the specified temperature in 10 min, and cooled down in 10 min. The temperature range was maintained with a temperature controller, ventilation was used to cool down the environment at the end of each day's heat treatment and the relative humidity of the rearing environment was controlled at around 50–60%. The birds in HT and HT + RSV groups received the basal diet and the basal diet with 500 mg/kg of RSV, respectively. RSV (>98% purity) was provided by the National Research Center of Engineering Technology for Utilization Ingredients from Botanicals. The trial period lasted for 14 days, from d 28 to 42 of the age of the broilers. The basal diet was formulated to meet the nutrient requirements of broilers based on the National Research Council (1994), and its nutrient profile was shown in Table 1.

**Table 1:** Composition and nutrient levels of the basal diets (air-dry basis, %).

**Item**	**Amount**	**Nutrient and energy content^**b**^**	
Ingredients		ME, kcal/kg	3,079.42
Corn	61.50	Crude protein	19.05
Soybean meal (46%)	28.00	Ca	0.90
Rice bran	3.00	Total phosphorus	0.42
Soybean oil	4.00	Available phosphorus	0.38
Dicalcium phosphate	1.00	Methionine	0.41
NaCl	0.20	Lysine	1.03
Sodium bicarbonate	0.18		
_L−_Lysine	0.10		
_DL−_Methionine	0.12		
Choline	0.10		
Limestone	1.30		
Premix^a^	0.50		
Total	100.00		

a *Supplied per kilogram of diet: 12,000 IU of vitamin A; 3,000 IU of vitamin D3; 26 IU of vitamin E; 2.2 mg of vitamin K3; 4 mg of vitamin B1; 10 mg of vitamin B2; 1.6 mg of vitamin B6; 15 mg of vitamin B12; 0.8 mg of D-biotin; 12.5 mg of D-Pantothenic acid; 1.2 mg of Folic acid; 32 mg of nicotinamide; 8 mg of Cu; 80 mg of Fe; 60 mg of Zn; 100 mg of Mn; 0.35 mg of I; 0.15 mg of Se*.

b *Calculated values*.

### Sample Collection

On d 7 and 14 of heat treatment, one broiler from each replicate was selected at random for blood sample collection from the wing vein and used to prepare the serum. On d 14 of heat treatment, one bird from each replication (*n* = 6/treatment) close to the average body weight of the group was killed by cervical dislocation for tissue samples collection. The about 2 cm jejunum and 1 g spleen were removed and kept in phosphate-buffered formalin (10%) for morphology analyses. The spleen, thymus, and bursa of Fabricius were removed and weighed to determine the growth index. About 2 g spleen of each sample was taken and immediately frozen in liquid nitrogen to analyze the cytokine levels, relative mRNA levels of immune signal pathway genes and apoptosis genes, and RNA-Seq and data analysis.

### Record of Growth Performance and Rectal Temperature

At d 28 and 42 old, all broilers were weighed individually. The feed intake per replicate was recorded daily. The average daily gain (ADG) and average daily feed intake (ADFI) were calculated in each replicate. The feed conversion ratio (FCR) was expressed as total feed intake/total body weight gain. One bird per replicate was randomly selected to measure rectal temperature using a digital Celsius thermometer at d 1 (time 09:00), d 1 (time 17:00), d 3 (time 17:00), d 8 (time 17:00), and d 14 (time 17:00) of heat stress, respectively.

### Measurement of Serum Hormone Concentration

The concentrations of serum corticosterone (CORT), adrenocorticotropic hormone (ACTH), triiodothyronine (T3), and thyroxine (T4) were measured using the commercial ELISA kits according to manufacturer's instructions (ZCIBIO Technology Co., Ltd., Shanghai, China). This assay had high sensitivity and excellent specificity for the detection of chicken CORT, ACTH, T3, and T4. No significant cross-reactivity or interference between chicken CORT, ACTH, T3, T4, or any other analogs was observed. The dynamic ranges of CORT, ACTH, T3, and T4 ELISA kits were 5 ng/mL−160 ng/mL, 2.5 pg/mL−80 pg/mL, 0.25 nmol/L−8 nmol/L, and 7.5 nmol/L−240 nmol/L, respectively. The sensitivity levels of CORT, ACTH, T3, and T4 ELISA kits were 1.0 ng/mL, 0.1 pg/mL, 0.1 nmol/L, and 1.0 nmol/L, respectively. To assess intraassay precision, 3 samples of a known protein concentration were tested 20 times on the same plate, and the CV was calculated to be <10%. Interassay precision was assessed using 20 assays of the 3 samples of known concentration, and the calculated CV was <13%. The microplates provided in these kits have been precoated with antibodies specific for chicken CORT, ACTH, T3, or T4. To quantitatively determine the levels of CORT, ACTH, T3, or T4 present in the samples, horseradish peroxidase-conjugated antibodies specific for CORT, ACTH, T3, or T4were added to each well. A standard curve was generated by plotting the optical density against the corresponding concentration of the standards and was subsequently used to determine the amounts of CORT, ACTH, T3, or T4 in an unknown sample.

### Jejunum Morphology Measurements

Formalin-fixed samples were dehydrated, embedded in paraffin, and the jejunum sections were stained with hematoxylin and eosin. The mucosa structure was observed with a BX43 Olympus microscope (Olympus, Tokyo, Japan) and analyzed with Motic images advanced 3.2 software system (CaseViewer, Xiamen, China). Ten well-orientated intact crypt–villi units per sample were selected for measurement, and villus height to crypt depth ratio (V/C) was calculated.

### Immune Organ Growth Index Determination

Immune organ index (%) was calculated as the immune organ fresh weight (g)/chicken weight (g) × 100% before slaughter (6).

### Splenic Cytokine Measurements

The concentrations of interleukin tuftsin, properdin, fibronectin (FN), interleukin (IL)-1β, IL-2, IL-4, tumor necrosis factor (TNF)-α, and interferon (IFN)-γ in the spleen of broilers were measured using commercially available ELISA kits specific for chicken, according to manufacturer's instructions (ZCIBIO Technology Co., Ltd., Shanghai, China).

### RNA Extraction, Reverse Transcription, and Real-Time PCR Analysis

To quantify mRNA, ~50 mg of spleen tissue was pulverized in liquid nitrogen, and total RNA was isolated from the homogenate using TRIzol (Beyotime Biotechnology, Shanghai, China) according to manufacturer instructions. The first-strand cDNA was then synthesized using a reverse transcription kit (TaKaRa, Japan). All primers were designed in NCBI using the chick gene sequence to produce an amplification product (Table 2). Real-time PCR was performed as described in a previous study (14). The relative expression of mRNA was calculated using the 2^−ΔΔCt^ method after normalization with GAPDH as a housekeeping gene.

**Table 2:** Sequence of primers for real-time PCR.

**Accession no**.	**Target gene^**a**^**	**Nucleotide sequence of primers(5'-3')**	**Product size (bp)**
NM_001006685.1	*HSP70*	F: CTGGCAATAAGCGAGCAGTGAGG	86
		R: AATGCTGGCTTGCGTGGAAGAG	
NM_205129	*NF-κB*	F: GTGTGAAGAAACGGGAACTG	205
		R: GGCACGGTTGTCATAGATGG	
NM_001001472.2	*IKB-α*	F: GGCAGATGTGAACAAGGTGA	118
		R: TATCTGCAGGTCAGCTGTGG	
NM_001353939.1	*p38 MAPK*	F: GCGAGTCCCTAATGCCTACG	199
		R: ACAACTGTTGAGCCACACTCA	
NM_205095.1	*JNK*	F: TGACCGAGTGAGGAGACGAT	211
		R: ACTGTATCGAACGCAGCACA	
NM_205388.1	*ERK*	F: AGAATCTCACAGCGTCTCGC	235
		R: GGTGTGATTCATCAGCATCTTCA	
NM-001199384.1	*MDM2*	F: CAAATGCAACTTCCCAGCCAAC	125
		R: GGTCAACGAGATGCTGTCCGAC	
NM_205264.1	*TP53*	F: CCCATCCTCACCATCCTTACA	108
		R: CTCGATCTTGCGGTCCCTC	
XM_416167.6	*Apaf-1*	F: AAGGGCATAAGGAAGCAATCAA	156
		R: CAGCACAAGAAAGAACAGCACC	
XM_015290060.2	*Bax*	F: TATGGGACACCAGGAGGGTA	166
		R: CGTAGACCTTGCGGATAAAGC	
XM_015288356.2	*Fas*	F: CTTCTCGGTGTG AACATTGCG	300
		R: GCTGGTGGGTCAGGTCAACATC	
NM_205339.2	*BCL-2*	F: ATCGTCGCCTTCTTCGAGTT	150
		R: ATCCCATCCTCCGTTGTCCT	
XM_015276122.2	*Caspase3*	F: TACCGGACTGTCATCTCGTTCAGG	166
		R: ACTGCTTCGCTTGCTGTGATCTTC	
XM_424580.6	*Caspase9*	F: CCGAAGGAGCAAGCACG	243
		R: AGGTTGGACTGGGATGGAC	
XM_015281453.2	*Cyt-C*	F: TGTCCAGAAATGTTCCCAGTGC	138
		R: CCTTTGTTCTTATTGGCATCTGTG	
NM_204305.1	*GAPDH*	F: CGATCTGAACTACATGGTTTAC	153
		R: TCTGCCCATTTGATGTTGC	
NM_205242.2	*LRP1*	F: CGAGTGCCAGAACCTGATGT	197
		R: ATTCGCAGCGCTCGATCTTA	
NM_001277704.1	*PERPB*	F: CCCTACGTGCACCAAGCATC	102
		R: TGCTCTCCCCCATCCATAGTC	
XM_015296060.2	*PIGs*	F: GAATGAAGGACTGGCCGACT	168
		R: GCCACTGCTCGGTATACCTCT	
NM_205508.1	*FOS*	F: GGGGACAGCCTCACCTACTA	84
		R: GGTGCAGAAATCCTGCGAGT	
NM_001024835.1	*IL-21*	F: AAAAGATGTGGTGAAAGATAAGGATGT	78
		R: GCTGTGAGCAGGCATCCA	
NM_001010842.2	*HSPB9*	F: AGAGACCATCTTCAGCGAGC	177
		R: TTCTTCACATCCTGGCAGACG	
NM_001307950.1	*URAH*	F: CACGATGGAGGTGAGGCAG	313
		R: GATGGTGAAGACGACCTCCA	
NM_001198975.1	*NRXN1*	F: AGCTCAGGTGGGTTAGCAAAT	349
		R: ACAAGCGTTCATTTAGATGTTGAGA	
NM_001318433.1	*MMR1L*	F: AGTGCATTCAGTCAGGTGGAA	264
		R: GGATGCTATTCCAGGACCCG	
NM_001006525.1	*EAF2*	F: TGGATGACATTGAACGAGAACTA	313
		R: CCAGATTCACTCAGCTGCAA	
XM_414168.6	*HSD17B2*	F: CGTGCTCATCACAGGAAGCG	112
		R: AGCTCCAGGGCCATCCTTAT	
XM_025155931.1	*BLB3*	F: TCCTGTGCAGTAAGAAAGGTCA	106
		R: TGCAGATTCTGGTTGGAGGC	
NM_001201386.1	*OC3*	F: ACTCCGAGTGTAGGGAGAGC	120
		R: AGGGCTCTTTGGGATCTTTCT	
NM_001030731.1	*CD36*	F: CTTTGCACCCATTGGACACT	123
		R: TCTTCGTGAGAGAAGCTGTATGG	

a *HSP70, heat shock protein 70; NF-κB, nuclear factor kappa-B; IKB-α, inhibitor of NF-κB-α; p38 MAPK, p38 mitogen-activated protein kinase; JNK, c-Jun N-terminal kinase; ERK, extracellular regulated protein kinases; MDM2, murine double minute 2; TP53, tumor protein p53; Apaf-1, apoptotic protease activating factor-1; Bax, BCL-2-associated X; Fas, Fas cell surface death receptor; BCL-2, B-cell lymphoma 2; Cyt-C, Cytochrome c; LRP1, LDL receptor related protein 1/CD91; PERPB, p53 apoptosis effector related to PMP22 B; PIGs, phosphatidylinositol glycan anchor biosynthesis class S; GAPDH, glyceraldehyde 3-phosphate dehydrogenase; FOS, Fos proto-oncogene, HSPB9, heat shock protein family B (small) member 9; BLB3, Major histocompatibility complex class II beta chain BLB3; EAF2, ELL associated factor 2; HSD17B2, Gallus gallus hydroxysteroid 17-beta dehydrogenase 2; IL21, interleukin 21; NRXN1, neurexin 1; OC3, osteocalcin-like protein OC3; CD36, CD36 molecule; MMR1L, macrophage mannose receptor 1-like*.

### TUNEL Staining

Spleen samples were pretreated as a method for H&E staining. The TUNEL staining was performed using a commercial kit (Servicebio, Wuhan, China). DNA breaks were detected on a single-cell level by terminal-deoxynucleotidyl transferase-mediated nick end labeling (TUNEL) with an *in situ* cell death detection kit (Servicebio, Wuhan, China) as described in a previous study (15).

### RNA-Seq and Data Analysis

Total RNA was isolated from the spleen using an RNA Isolated Kit (TaKaRa, Japan) according to the manufacturer's protocol. The quality and quantity of extracted RNA were determined using Nanodrop Spectrophotometers (IMPLEN, CA, USA) and a Qubit Fluorometer (Life Technologies, CA, USA). RNA Integrity Number (RIN) was determined using 2,100 RNA Nano 6,000 Assay Kit (Agilent Technologies, CA, USA). The prepared libraries were sequenced using an Illumina HiSeq3000 sequencer (SeqHealth Tech, Wuhan, China). Subsequent procedures were undertaken as described previously (16).

After the quality raw RNA-seq data was controlled, we obtained clean data for further analysis. Read count matrices were obtained using the Feature Counts package, the data were aligned to reference chicken genome (GRCg6a) downloaded from NCBI (https://www.ncbi.nlm.nih.gov/genome/111?genome_assembly_id=451987) performed by HISAT2. An FDR corrected *P*-value cutoff of 0.05 and a fold-change cut-off of 2, were used as the standard for differentially expressed genes using the edge R package (version 3.12.1). The gene ontology database (http://www.geneontology.org/) was used to determine GO functional classification and the KEGG pathway database (http://www.genome.jp/kegg/) was used to determine KEGG pathway involvement of the differentially expressed genes using the KOBAS software (version: 2.1.1) with a corrected *P*-value cutoff of 0.05 to judge statistically significant enrichment.

### Statistical Analyses

All statistical analyses except the RNA-Seq were performed using SPSS 22.0 software and shown as mean ± standard error of the mean (SEM). The data were checked for normality and homoscedasticity of the data variance using the Shapiro-Wilk test and Levene's test, respectively. The parametric data were analyzed by 2-way ANOVA using the General Linear Models procedure. The model included the main effects of RSV, heat stress, and their interaction. Group comparisons were performed using the one-way ANOVA followed by Duncan's multiple-ranges test. The non-parametric data were analyzed by Kruskal–Wallis test followed by Dunn's multiple comparisons test (17). *P* values <0.05 were considered statistically significant.

## Results

### Growth Performance and Rectal Temperature

**Figure 1:**
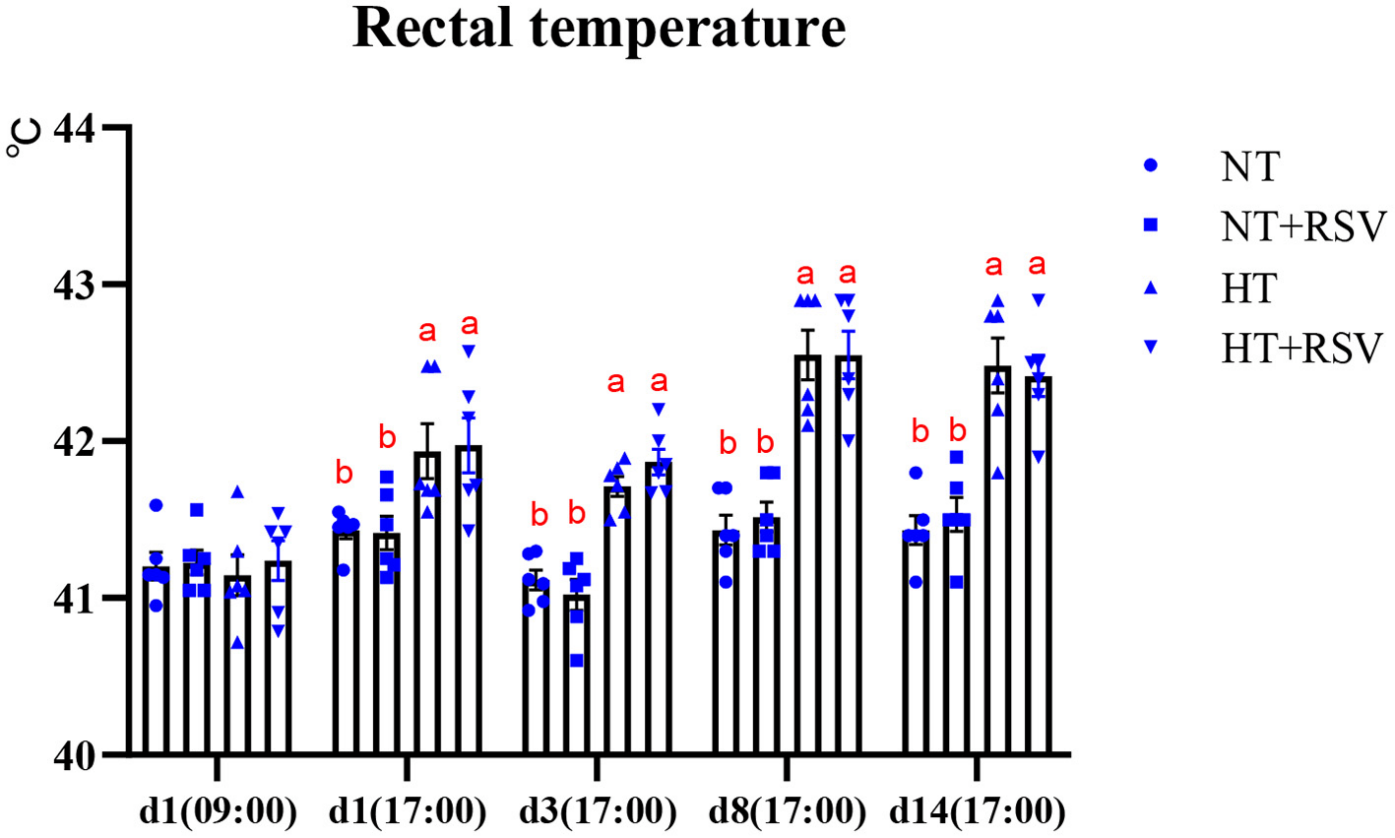
Effect of heat stress and RSV on rectal temperature of 42-day-old broilers (d 14 of heat treatment). Symbols and error bars represent means standard error. Bars represent the means ± SEM (*n* = 6), bars with different letters are statistically significant in different treatments. RSV, resveratrol; NT, normal temperature; HT, heat stress.

Heat stress significantly decreased the body weight (BW), ADG, and ADFI (*P* = 0.003, 0.001, and <0.001, respectively) (Table 3). Dietary RSV supplementation improved the FCR (*P* = 0.014), and partially reversed the negative alterations in BW, ADG, and ADFI caused by heat stress (*P* > 0.05). Dietary supplementation with RSV under heat stress increased BW and ADG by 2.03 and 4.08% compared with the HT group (*P* > 0.05). The FCR in the NT + RSV and HT + RSV groups decreased by 6.78 and 5.08% than the HT group (*P* < 0.05). The rectal temperature of birds at different time points was shown in Figure 1. At d 1 (09:00), before high-temperature exposure, all treatments had similar rectal temperatures, which increased during heat stress. However, RSV treatment had a non-significant effect on rectal temperature raised by heat stress.

**Table 3:** Effect of heat stress and RSV on growth performance of 42-day-old broilers (14 of heat treatment).

	**Dietary treatments**				**SEM**	***P*** **-values**		
	**NT**	**NT + RSV**	**HT**	**HT + RSV**		***P*-RSV**	***P*-Stress**	***P*-RSV × stress**
BW, g	984.52^a^	987.55^a^	929.82^b^	948.73^ab^	8.187	0.435	0.003	0.571
ADG, g	31.04^a^	31.02^a^	27.47^b^	28.59^b^	0.484	0.486	0.001	0.471
ADFI, g	70.24^a^	68.26^a^	64.76^b^	63.96^b^	0.749	0.232	<0.001	0.604
FCR	2.26^ab^	2.20^b^	2.36^a^	2.24^b^	0.019	0.014	0.051	0.424

*Data are means of 6 replicates of 18 broilers per dietary treatment. Data are presented as mean ± SEM. Values in the same row with different superscript are significantly different by one-way ANOVA. P-values (P-RSV, P-Stress, or P-RSV × Stress) ≤ 0.05 indicates RSV, heat stress, or the interaction between heat stress and RSV. RSV, resveratrol; BW, body weight; ADG, average daily gain; ADFI, average daily feed intake; FCR, feed conversion ratio; NT, normal temperature; HT, heat stress*.

### Serum Hormone Concentration

RSV supplementation significantly decreased the content of ACTH on d 7 (*P* < 0.001) and 14 (*P* = 0.020), and CORT on d 14 (*P* = 0.001) (Table 4). Heat stress significantly increased CORT, ACTH, T3, and T4 concentrations in serum on d 7 and 14 (*P* < 0.01). Whereas, the combination RSV and heat stress markedly decreased ACTH and T3 on d 7 (*P* = 0.023 and 0.049, respectively), and CORT on d 14 (*P* = 0.014).

**Table 4:** Effect of heat stress and RSV on the serum hormone concentration of 35-day-old and 42-day-old broilers (d 7 and 14 of heat treatment).

	**Dietary treatments**				**SEM**	***P*** **-values**		
	**NT**	**NT + RSV**	**HT**	**HT + RSV**		***P*-RSV**	***P*-stress**	***P*-RSV × stress**
**D7**								
CORT, ng/mL	67.43^b^	52.99^b^	112.43^a^	108.78^a^	6.55	0.163	<0.001	0.396
ACTH, pg/mL	43.30^bc^	37.19^c^	63.67^a^	46.33^b^	2.48	<0.001	<0.001	0.023
T3, nmol/L	4.10^c^	4.55^bc^	6.62^a^	5.37^b^	0.29	0.329	0.001	0.049
T4, nmol/L	125.00^bc^	111.18^c^	192.66^a^	158.92^ab^	9.36	0.084	<0.001	0.452
T3/T4	0.033	0.042	0.035	0.035	0.0017	0.199	0.514	0.221
**D14**								
CORT, ng/mL	51.47^bc^	45.39^c^	103.64^a^	65.36^b^	5.487	0.001	<0.001	0.014
ACTH, pg/mL	35.66^c^	34.30^c^	61.29^a^	48.33^b^	2.637	0.020	<0.001	0.055
T3, nmol/L	3.88^b^	3.73^b^	5.27^a^	4.39^ab^	0.191	0.106	0.003	0.251
T4, nmol/L	104.23^b^	107.96^b^	156.96^a^	143.65^a^	6.028	0.558	<0.001	0.302
T3/T4	0.038	0.036	0.034	0.031	0.0015	0.366	0.152	0.952

*Data are presented as mean ± SEM, n = 6. Values in the same row with different superscript are significantly different by one-way ANOVA. P-values (P-RSV, P-Stress, or P-RSV × Stress) ≤ 0.05 indicates RSV, heat stress, or the interaction between heat stress and RSV. RSV, resveratrol; CORT, corticosterone; ACTH, adrenocorticotropic hormone; T3, triiodothyronine; T4, thyroxine; NT, normal temperature; HT, heat stress*.

The concentrations of CORT, ACTH, T3, and T4 in serum were higher (*P* < 0.05) in the HT group than the NT and NT + RSV groups on d 7 and 14, whereas supplementation with RSV under heat stress markedly decreased the concentration of ACTH on d 7 and 14, T3 on d 7, and CORT on d 14 (*P* < 0.05). However, supplementation with RSV and the combination of RSV and stress did not affect (*P* > 0.05) the contents of T4 and the ratio of T3–T4 (T3/T4) on d 7 and 14.

### Morphology of Jejunum and Growth Index of the Thymus, Spleen, and Bursa of Fabricius

Dietary supplementation with RSV significantly increased the V/C value in the jejunum (*P* = 0.02) (Table 5). The V/C value was higher in the HT + RSV and NT + RSV groups than in the HT group (*P* < 0.05).

**Table 5:** Effect of heat stress and RSV on the morphology of jejunum of 42-day-old broilers (d 14 of heat treatment).

	**Dietary treatments**				**SEM**	***P*** **-values**		
	**NT**	**NT + RSV**	**HT**	**HT + RSV**		***P*-RSV**	***P*-stress**	***P*-RSV × stress**
Villus height, μm	1,039.17	1,080.25	994.48	1,090.73	28.800	0.268	0.779	0.651
Crypt depth, μm	161.46	157.90	188.41	156.94	5.648	0.077	0.222	0.077
V/C	6.45^ab^	6.83^a^	5.40^b^	6.99^a^	0.226	0.020	0.309	0.080

*Data are presented as mean ± SEM, n = 6. Values in the same row with different superscript are significantly different by one-way ANOVA. P-values (P-RSV, P-Stress, or P-RSV × Stress) ≤ 0.05 indicates RSV, heat stress, or the interaction between heat stress and RSV. RSV, resveratrol; V/C, Villus length/Crypt depth; NT, normal temperature; HT, heat stress*.

Heat stress, the supplementation with RSV, and the combination of RSV and stress did not affect the growth index of the spleen, thymus, and bursa of Fabricius on d 14 (*P* > 0.05) (Table 6).

**Table 6:** Effect of heat stress and RSV on organ index of 42-day-old broilers (d 14 of heat treatment).

	**Dietary treatments**				**SEM**	***P*** **-values**		
	**NT**	**NT + RSV**	**HT**	**HT + RSV**		***P*-RSV**	***P*-stress**	***P*-RSV × stress**
Spleen, %	0.326	0.251	0.196	0.176	0.0193	0.511	0.096	0.743
Thymus, %	0.322	0.349	0.352	0.300	0.0107	0.796	0.204	0.064
Bursa of Fabricius, %	0.087	0.121	0.139	0.121	0.0121	0.861	0.173	0.504

*Data are presented as mean ± SEM, n = 6. Values in the same row with different superscript are significantly different by one-way ANOVA. P-values (P-RSV, P-Stress, or P-RSV × Stress) ≤ 0.05 indicates RSV, heat stress, or the interaction between heat stress and RSV. RSV, resveratrol; NT, normal temperature; HT, heat stress*.

### The Levels of Cytokines in the Spleen

The supplementation with RSV had significant effect on the levels of IL-1β, TNF-α, and IFN-γ (*P* = 0.024, 0.039, and 0.011, respectively) (Table 7). Heat stress markedly elevated the levels of tuftsin (*P* = 0.001), properdin (*P* < 0.001), FN (*P* < 0.001), IL-1β (*P* < 0.001), IL-2 (*P* < 0.001), IL-4 (*P* < 0.001), and TNF-α (*P* < 0.001), whereas it decreased IFN-γ levels (*P* = 0.002). Whereas, the combination of RSV and heat stress markedly decreased tuftsin (*P* = 0.031), properdin (*P* = 0.021), IL-1β (*P* = 0.001), IL-2 (*P* = 0.016), IL-4 (*P* = 0.012), and TNF-α (*P* = 0.038). Dietary supplementation with RSV under heat stress lowered the concentration of tuftsin, properdin, FN, IL-1β, IL-2, IL-4, and TNF-α, but elevated IFN-γ compared with the HT group (*P* < 0.05).

**Table 7:** Effect of heat stress and RSV on the levels of cytokines in spleen of 42-day-old broilers (d 14 of heat treatment).

	**Dietary treatments**				**SEM**	***P*** **-values**		
	**NT**	**NT + RSV**	**HT**	**HT + RSV**		***P*-RSV**	***P*-stress**	***P*-RSV × stress**
Tuftsin, ng/g	75.81^b^	86.32^b^	122.38^a^	97.47^b^	5.127	0.354	0.001	0.031
Properdin, ng/g	36.28^c^	45.33^c^	87.53^a^	70.79^b^	5.038	0.459	<0.001	0.021
FN, μg/g	455.96^b^	424.82^b^	711.81^a^	561.30^b^	33.610	0.087	0.001	0.250
IL-1β, pg/g	1,533.07^c^	1,964.51^c^	4,875.27^a^	3,011.86^b^	310.940	0.024	<0.001	0.001
IL-2, pg/g	1,196.72^c^	1,465.20^bc^	2,284.60^a^	1,792.11^b^	110.252	0.443	<0.001	0.016
IL-4, pg/g	550.15^c^	648.47^c^	1,189.15^a^	891.75^b^	62.983	0.177	<0.001	0.012
TNF-α, pg/g	238.34^c^	238.95^c^	527.82^a^	373.31^b^	30.494	0.039	<0.001	0.038
IFN-γ, pg/g	491.59^a^	587.17^a^	313.52^b^	456.98^a^	28.859	0.011	0.002	0.576

*Data are presented as mean ± SEM, n = 6. Values in the same row with different superscript are significantly different (P < 0.05) by one-way ANOVA. P-values (P-RSV, P-Stress, or P-RSV × Stress) ≤ 0.05 indicates RSV, heat stress, or the interaction between heat stress and RSV. RSV, resveratrol; NT, normal temperature; HT, heat stress; FN, fibronectin; IL-1β, Interleukin-1β; IL-2, Interleukin-2; IL-4, Interleukin-4; TNF-α, tumor necrosis factor-α; IFN-γ, interferon γ*.

### Levels of Apoptosis of the Spleen

**Figure 2:**
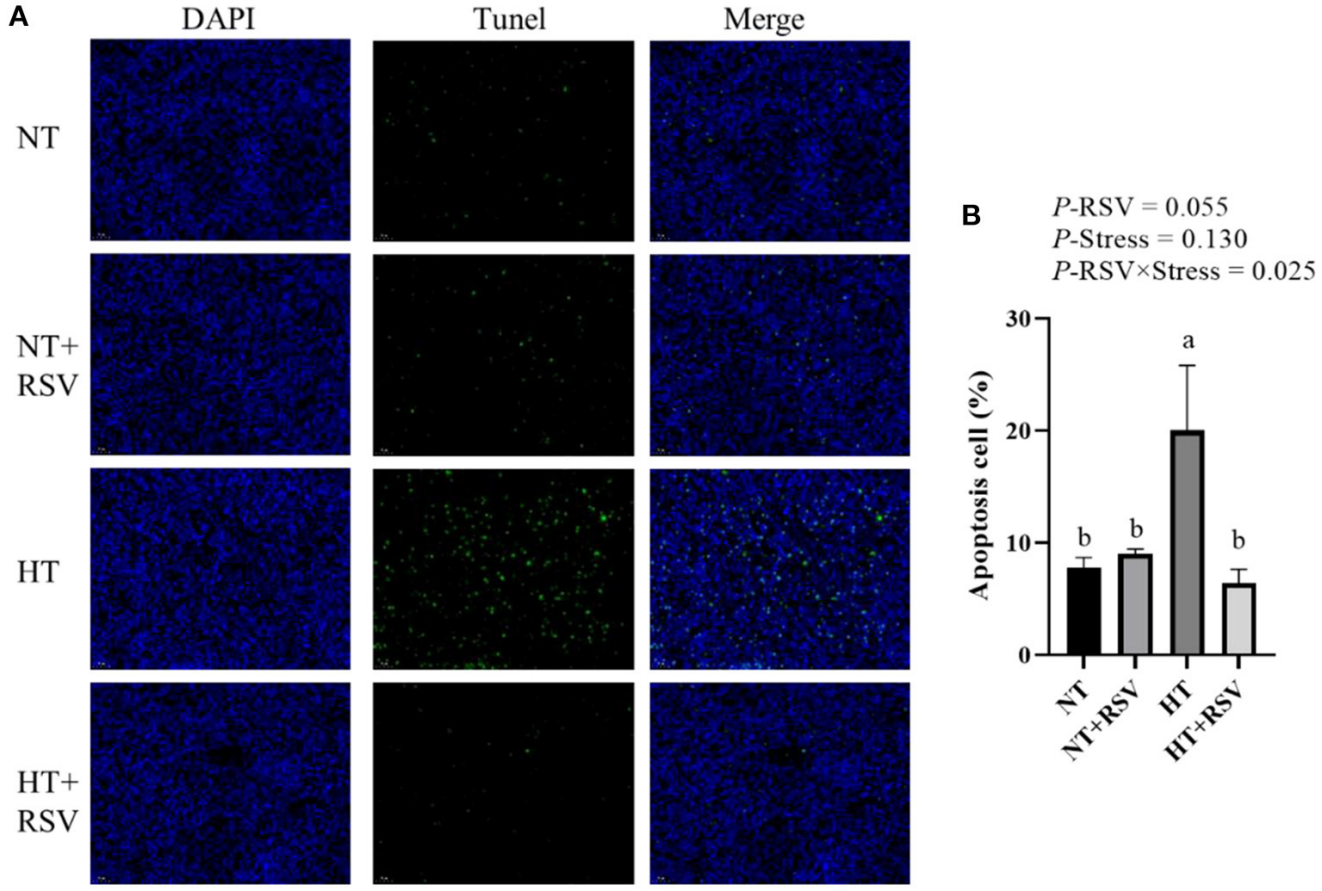
The frequency of apoptosis in spleens of 42-day-old broilers (d 14 of heat treatment) detected by TUNEL assay. **(A)** TUNEL staining. The blue fluorescence represents the nucleus, whereas the green fluorescence represents apoptotic cells. The magnification is ×400. **(B)** The percentage of the apoptotic cells. Symbols and error bars represent means standard error. Bars represent the means ± SEM (*n* = 6), bars with different letters are statistically significant in different treatments. *P*-values (*P*-RSV, *P*-Stress, or *P*-RSV × Stress) ≤ 0.05 indicates RSV, heat stress, or the interaction between heat stress and RSV. RSV, resveratrol; NT, normal temperature; HT, heat stress.

The TUNEL assay was used to detect the number of apoptotic cells in the spleen of the broiler. The TUNEL positive signal (green fluorescence) and nuclear signal (blue fluorescence) of the spleen were observed. As presented in Figures 2A,B, the combined RSV and heat stress markedly decreased apoptotic cells (*P* = 0.025). The number of apoptotic cells was significantly higher in the HT-treated group than in the other groups (*P* < 0.05). RSV supplementation during heat stress markedly reduced the frequency of apoptotic cells compared to the heat stress-treated group (*P* < 0.05).

**Figure 3:**
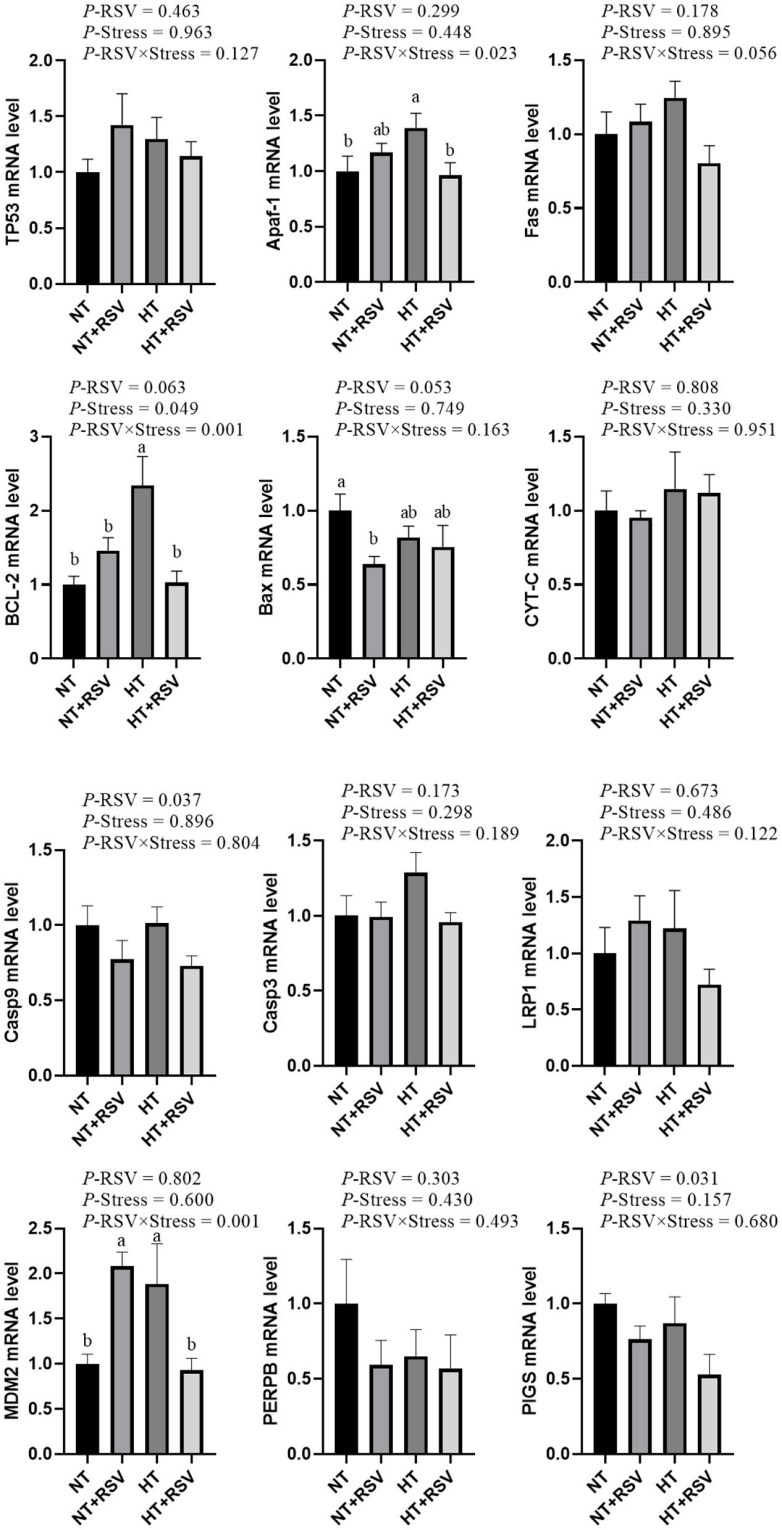
Effect of heat stress and RSV on some related gene involved in apoptosis genes expression levels in spleen of 42-day-old broilers (d 14 of heat treatment). Bars represent the means ± SEM (*n* = 6), bars with different letters are statistically significant in different treatments. *P*-values (*P*-RSV, *P*-Stress, or *P*-RSV × Stress) ≤ 0.05 indicates RSV, heat stress, or the interaction between heat stress and RSV.

Based on the above results, the relative mRNA levels of apoptosis genes in the spleen of the broilers were investigated. As shown in Figure 3, the supplementation with RSV decreased the relative gene expression of *Caspase 9* and (phosphatidylinositol glycan anchor biosynthesis class S) *PIGs* (*P* = 0.037 and 0.031, respectively). Heat stress significantly affected the relative gene expression of B-cell lymphoma 2 (*BCL-2*) (*P* = 0.049). The relative gene expression of *BCL-2* (*P* = 0.001), apoptotic protease activating factor-1 (*Apaf-1*) (*P* = 0.023), and murine double minute 2 (*MDM2*) (*P* = 0.001) were markedly decreased by the combination of RSV and heat stress.

As expected, the relative mRNA levels of *BCL-2* and *Apaf-1* in the spleen were elevated in the HT group compared with the NT group (*P* < 0.05). Compared with the NT group, the relative mRNA level of *MDM2* was markedly increased in the HT and NT+RSV groups (*P* < 0.05), and BCL-2-associated X (*BAX*) level was reduced in the NT + RSV group (*P* < 0.05). Remarkably, the RSV supplement under heat stress down-regulated the splenic *BCL-2, Apaf-1*, and *MDM2* mRNA expression compared with the HT group (*P* < 0.05).

### Transcriptional Response in Broilers Spleen After RSV Treatment Under Heat Stress

**Figure 4:**
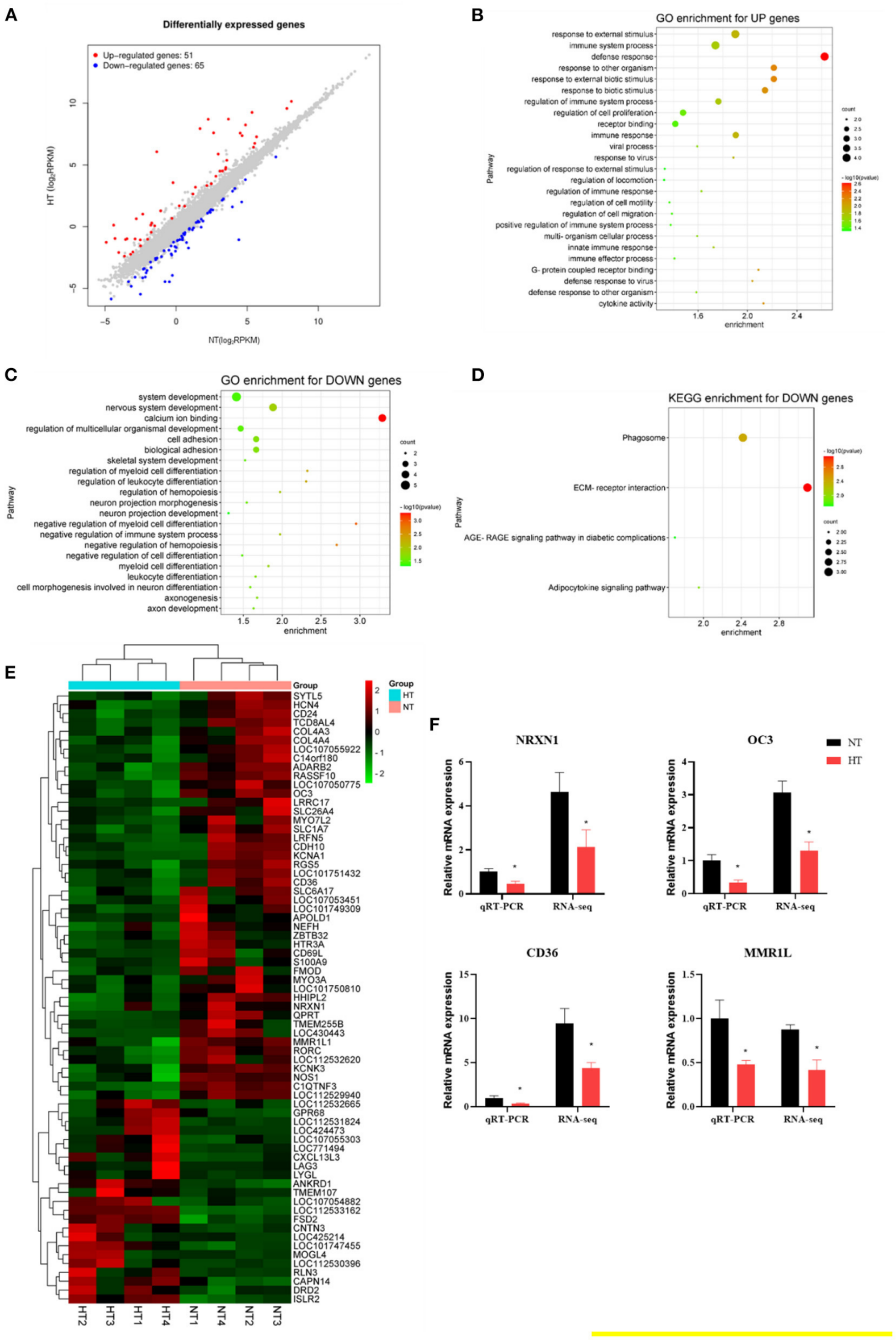
Effects of heat stress on the splenic transcriptome of 42-day-old broilers (d 14 of heat treatment). **(A)** Numbers of heat stress-induced differentially expressed splenic genes (*P* < 0.05 and fold change ≥ 2; *n* = 4). **(B)** GO terms enrichment of splenic gene expression for up genes in broilers (*P* < 0.05). **(C)** GO terms enrichment of splenic gene expression for down genes in broilers (*P* < 0.05). **(D)** KEGG terms enrichment of splenic gene expression for down genes in broilers (*P* < 0.05). **(E)** The heat map of DEG genes in broilers. **(F)** Validation of the gene expression profile by real-time PCR. Asterisk means significant difference with NT group (*P < 0.05). RSV, resveratrol; NT, normal temperature; HT, heat stress.

The splenic transcriptome showed that 65 genes were downregulated and 51 genes were upregulated in the HT group compared to the NT group (fold-change of at least 2) (Figures 4A,E). The upregulated genes enriched GO terms were involved in defense response, response to external biotic stimulus, cytokine activity, regulation of immune system process, innate immune response (Figure 4B). The downregulated genes involved calcium ion binding, negative regulation of immune system process, and cell adhesion (Figure 4C). KEGG analysis revealed a reduction in pathways associated with ECM-receptor interaction, phagosome, and adipocytokine signaling pathway (Figure 4D). The expression of four downregulated [*NRXN1* (neurexin 1), *OC3* (osteocalcin-like protein OC3)*, CD36* (CD36 molecule), and *MMR1L* (macrophage mannose receptor 1-like)] genes was evaluated by RT-PCR analysis. The results showed similar expression patterns observed with transcriptome sequencing, validating our transcriptome data (Figure 4F).

**Figure 5:**
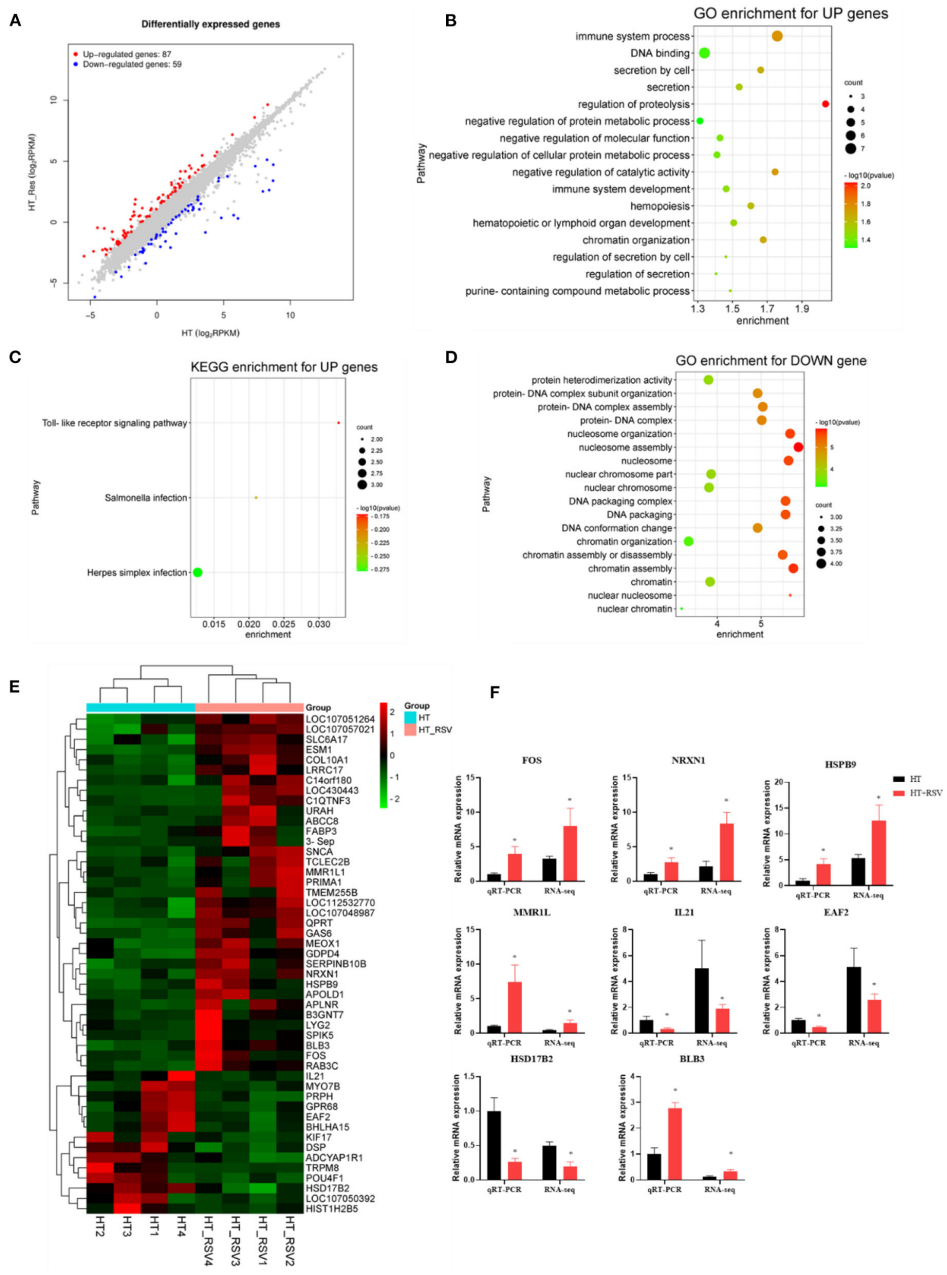
Effects of heat stress and RSV on the splenic transcriptome of 42-day-old broilers (d 14 of heat treatment). **(A)** Numbers of RSV-induced differentially expressed splenic genes (*P* < 0.05 and fold change ≥ 2; *n* = 4). **(B)** GO terms enrichment of splenic gene expression for up genes in broilers (*P* < 0.05). **(C)** KEGG terms enrichment of splenic gene expression for up genes n broilers (*P* < 0.05). **(D)** GO terms enrichment of splenic gene expression for down genes in broilers (*P* < 0.05). **(E)** The heat map of DEG genes in broilers. **(F)** Validation of the gene expression profile by real-time PCR. Asterisk means significant difference with HT group (^*^P < 0.05). RSV, resveratrol; HT, heat stress.

We further investigated the splenic transcriptome of the broilers in the HT and HT + RSV groups, 59 genes were downregulated and 87 genes were upregulated in the HT + RSV group compared to the HT group (fold-change of at least 2) (Figures 5A,E). The upregulated genes enriched GO terms were involved in positive regulation of immune system process, hematopoietic or lymphoid organ development, immune system development, and hemopoiesis (Figure 5B). KEGG enrichment for up genes showed an increase in pathways associated with the toll-like receptor signaling pathway and salmonella infection (Figure 5C). The downregulated genes were involved in protein heterodimerization activity, protein-DNA complex subunit organization (Figure 5D). The expression of five upregulated [*FOS* (Fos proto-oncogene), *NRXN1, HSPB9* (heat shock protein family B (small) member 9), *MMR1L*, and *BLB3* (Major histocompatibility complex class II beta chain BLB3)] and three downregulated [*EAF2* (ELL associated factor 2), *HSD17B2* (Gallus gallus hydroxysteroid 17-beta dehydrogenase 2), and *IL21* (interleukin 21)] genes were detected by RT-PCR analysis. The results showed similar expression patterns observed with transcriptome sequencing, validating our transcriptome data (Figure 5F).

### The Relative mRNA Levels of Immune Signal Pathway Genes in the Spleen of Broilers

**Figure 6:**
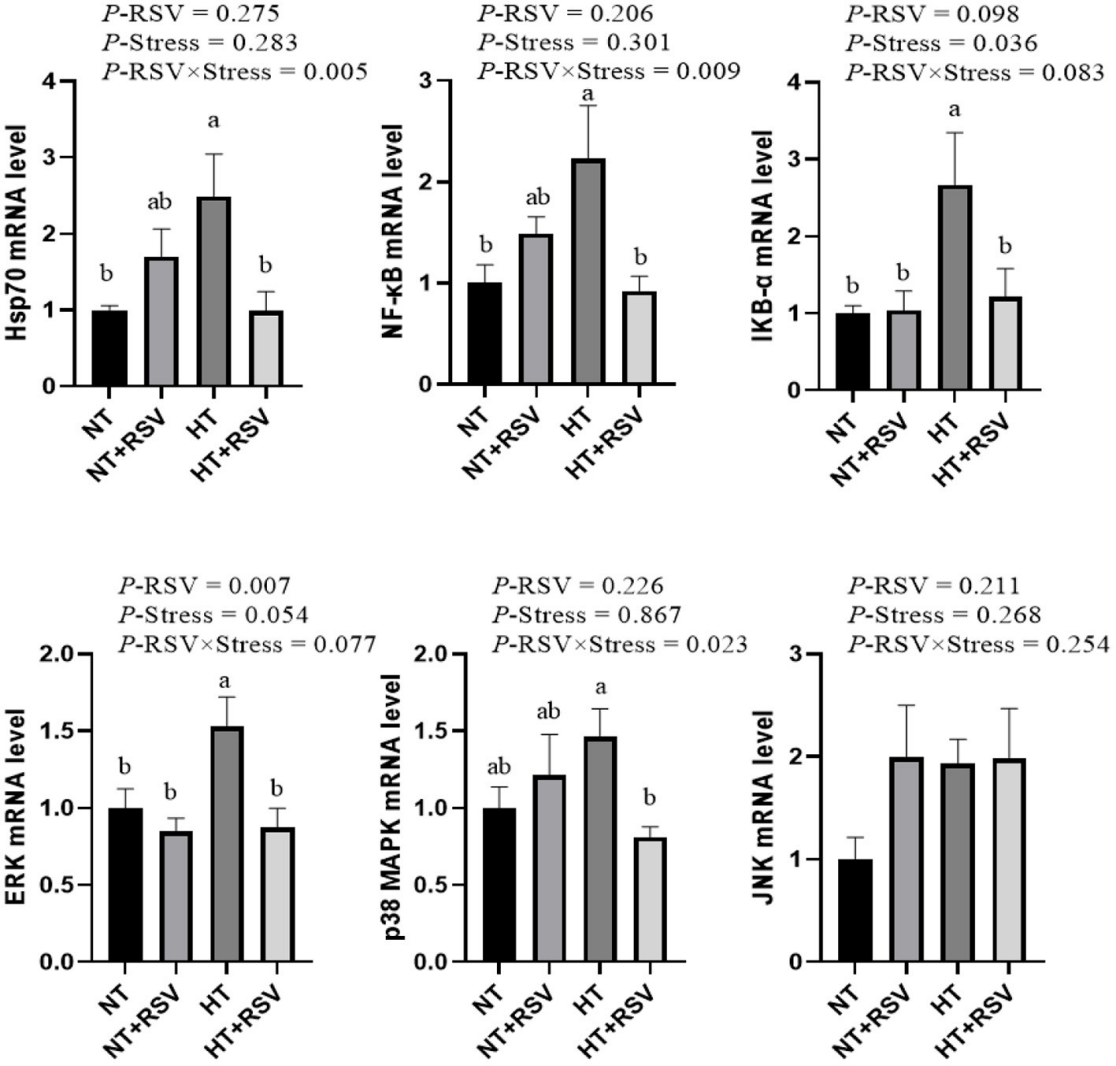
Effect of heat stress and RSV on immune signal pathway genes expression levels in spleen of 42-day-old broilers (d 14 of heat treatment). Bars represent the means ± SEM (*n* = 6), bars with different letters are statistically significant in different treatments. *P*-values (*P*-RSV, *P*-Stress, or *P*-RSV × Stress) ≤ 0.05 indicates RSV, heat stress, or the interaction between heat stress and RSV. RSV, resveratrol; NT, normal temperature; HT, heat stress.

Based on the above results, the relative genes expression of immune signal pathway genes in the spleen of broilers was investigated. As shown in Figure 6, the supplementation with RSV decreased the relative gene expression of extracellular regulated protein kinases (*ERK*) (*P* = 0.007). Heat stress increased the relative gene expression of inhibitor of NF-κB-α (*IKB-*α) (*P* = 0.036). The relative mRNA expression of *NF-*κ*B* (*P* = 0.009), heat shock protein 70 (*HSP70*) (*P* = 0.005), and *p38 MAPK* (*P* = 0.023) were markedly decreased by the combination of RSV and heat stress.

The relative mRNA levels of *ERK* and *IKB-*α in the spleen were higher in the HT group than the NT and NT+RSV groups (*P* < 0.05), and *NF-*κ*B* and *HSP70* in the spleen were higher in the HT group than the NT group (*P* < 0.05). The HT + RSV group significantly down-regulated the *NF-*κ*B, ERK, IKB-*α, *HSP70*, and *p38 MAPK* mRNA levels in the spleen compared with the HT group (*P* < 0.05).

## Discussion

In this study, broilers reared under heat stress showed an increased rectal temperature from time point 2 to the end of the heat stress. The increase in rectal temperature justifies the plausibility of the chronic heat stress model (18). Similarly, many studies have reported that chronic heat exposure resulted in markedly higher rectal temperatures in the heat stress group of broilers (19, 20). Moreover, a previous study showed that diets supplemented with 500 mg/kg of RSV suppressed the increase in rectal temperature caused by cyclic heat stress (21). However, in this study, dietary RSV did not alleviate the increase in rectal temperatures in chronically heat-stressed broilers. The differences in thermoregulatory effects might be associated with species, doses of RSV, ages of animals, etc.

Heat stress decreased BW, ADG, and ADFI in this study while increasing FCR, which was consistent with earlier research (12, 19, 21). The retarded growth performance could be attributed to poor appetite, less efficiency of nutrients' absorption and utilization, and compromised health status, including endocrine disorders, systemic immune dysregulation, and oxidative damage (22). The supplementation with RSV has been demonstrated to increase ADG and decrease FCR during heat stress (21). Similarly, the present study found that dietary supplementation with RSV 500 mg/kg under heat stress enhanced BW and ADG by 2.03 and 4.08 percent, respectively, compared to the HT group. The FCR in the NT+RSV and HT + RSV groups decreased by 6.78 and 5.08% than the HT group. It might be explained that RSV induced the secretion of hormones (such as CORT and ACTH) and altered intestinal surface area to resist the damage caused by heat stress, to improve the production performance and feed conversion rate.

Increased blood concentrations of CORT are assumed to indicate heat stress. Chronic heat stress increased the concentration of CORT and ACTH in serum (21). This was consistent with our study. CORT has been shown in studies of mammals to regulate appetite via the effects of neuropeptide Y and leptin (23). Glucocorticoids reduced the number of lymphocytes in the blood during stress by inhibiting the maturation of their precursor cells in the thymus and inducing apoptosis, thereby suppressing the animal's immune system (24).

Besides the adrenal hormones, thyroid hormones are also important for maintaining homeostatic in animals (25). On d 7 and 14 of heat stress, the levels of T3 and T4 in serum in group HT were considerably higher than in the NT and NT + RSV groups, showing that a stressor could rapidly affect the regulation of the hypothalamic-pituitary-thyroid (HPT) axis. Similarly, chronic mild stress increased T4 and T3 serum levels in two different strains of rats (26). Inconsistently, chronic heat stress has been found to increase plasma concentration of CORT with a concomitant reduction in circulating thyroid hormones levels (27, 28). Our previous results indicated that heat stress significantly lowered the concentrations of T3 and T3/T4, and had a varying effect on the contents of T4 (21). A study showed that heat stress markedly reduced plasma T3 and T3/T4, and increased T4 in laying hens (29). Stress, depending on the type of stress had a complex influence on the HPT axis, which was affected by stress duration, intensity, predictability, biofluids, and hormone sampling time (26, 30).

Dietary RSV supplementation has been shown to reduce serum CORT and protect broilers from heat stress (31), which was consistent with the findings of this investigation. This study showed that supplementation with RSV under heat stress markedly decreased the concentration of ACTH on d 7 and CORT on d 14 when compared with the HT group. RSV may have weakened the hypothalamic-pituitary-adrenal (HPA) axis and inhibited adrenocortical hydroxylase. Furthermore, the HT+ RSV group had lower T3 concentrations on d 7 than the HT group, although supplementation with RSV and the combination of RSV and stress did not affect T4 and T3/T4 concentrations on d 7 and 14. RSV has previously been found to reduce HPA axis hyperactivity and modulate the HPT axis, alleviating depression in a stressed rat model (32). This implied that RSV supplementation had a positive regulatory effect on abnormal levels of serum hormones during heat stress and was considered essential evidence to support changes in growth performance, rectal temperature, and anti-inflammatory capacity. In addition, increased blood CORT concentrations induced by heat stress have been shown to suppress the production of cytokines such as IL-4, IL-5, IL-6, IL-12, IFN-γ, and TNF-α (33). Glucocorticoids affected the balance of T-helper 1 (Th1) and T-helper 2 (Th2) by inhibiting IL-12.

It has been shown that heat stress could elevate the expression of pro-inflammatory cytokines and inhibit anti-inflammatory cytokines in broilers (17). A study exhibited that consecutive cyclic heat stress markedly elevated the hepatic mRNA levels of HSP70, TNF-α, and inducible nitric oxide synthase (34). Also, heat stress enhanced TNF-α and IL-4 relative mRNA levels in the chicken spleen while decreasing IFN-γ and IL-2 mRNA levels (35). The level of IFN-γ in the spleen of heat-stressed broilers was reported to be decreased (36). According to a prior study, the splenic relative mRNA levels of IL-1β, IL-4, and IL-6 were dramatically up-regulated, while IL-2 mRNA levels were significantly down-regulated in the heat stress-treatment chicken (37). High environmental temperatures contributed to a significant increase in the plasma contents of IL-1, IL-6, and TNF-α, leading to a further increase in the body temperature (38). This study showed that heat stress markedly elevated the levels of tuftsin, properdin, FN, IL-1β, IL-2, IL-4, and TNF-α in the spleen, whereas decreased IFN-γ levels. Lymphocytes are classified into 2 types, Th1 and Th2. In general, cytokines of the Th1-type (IL-2, IL-8, TNF-α, and IFN-γ) enhance cellular immunity, whereas cytokines of the Th2-type (IL-4, IL-6, and IL-10) act in humoral immunity (39, 40). Heat stress affected the immune system by altering the Th1:Th2 ratio (6), dietary supplementation with RSV might maintain the Th1: Th2 balance.

The basic function of tuftsin is to promote phagocytosis and regulate immune function (41). Serum tuftsin levels in patients with impaired spleen function decrease and lead to a decrease in neutrophil activity and increase in the incidence of infection (42). Properdin, a plasma glycoprotein, is the only known positive regulator in the complement system. It binds and stabilizes the alternative pathway C3 convertase C3bBb and may serve as a platform to form new C3bBb convertases on the cell surface (43–45). FN acts as a recognition factor for macrophages, and targets phagocytosed by macrophages must first bind to FN. Therefore, when FN decreases in the body, phagocytosis diminishes (46). In this study, dietary RSV supplementation significantly reduced the splenic levels of tuftsin, properdin, and FN caused by heat stress, implying that RSV supplementation improved the anti-inflammatory potential in white feather broilers.

Heat stress has been demonstrated that could cause apoptosis in animals (47–49). For instance, TUNEL staining revealed that heat-treated rats showed stronger villi epithelial cells undergoing apoptosis than the normal control (50). The increase in the apoptotic rate with increasing temperatures and apoptosis induction was an indication of the upregulation of cleaved caspase-3 (49). In line with those findings, the current investigation found that heat stress treatment significantly increased the frequency of apoptosis in splenic cells. The relative gene expression of *BCL-2* and *MDM2* was likewise elevated by heat stress. In response to cellular stressors, the p53 tumor suppressor triggered anti-proliferative mechanisms (51). *MDM2* is considered an oncogene due to the ability of its product to inhibit p53 tumor suppressor function (52). The *MDM2* gene is a downstream target of p53 and forms a tight auto-regulatory feedback loop. Inhibition of the *MDM2*-p53 feedback loop is essential for p53 activation in the cellular stresses response (53). Heat stress significantly enhanced the relative gene expression of *MDM2*, indicating *MDM2* activation may cause apoptosis.

It was reported that RSV has antagonistic effects on heat stress in chicken immune organs (6, 54). Consistent with the above studies, this study suggested that RSV markedly reduced the frequency of heat stress-induced apoptotic cells and enhanced immune function. The beneficial effects of RSV on cisplatin-induced oxidative stress, inflammation, and apoptosis in female rats' ovarian and uterine tissues have been discovered (55). RSV may alleviate hyperoxia-induced mitochondrial dysfunction and apoptosis in alveolar epithelial cells through the SIRT1/PGC-1α signaling pathway (56). Interestingly, in the present study, NT + RSV markedly decreased the relative gene expression of *BAX* compared with the NT group. The HT + RSV supplement down-regulated the *BCL-2* and *MDM2* mRNA levels in the spleen compared with the HT group. Caspase 3 plays an integral part in the progression of mitochondrial damage, which is positively associated with the apoptosis rate (57). As two members of the BCL-2 family, Bax exerts a pro-apoptotic effect, and BCL-2 plays an anti-apoptotic role, but both are critical regulators of apoptosis (58, 59). Therefore, the HT + RSV supplement down-regulated the *MDM2* mRNA levels compared with the HT group, suggesting that RSV can alleviate the apoptosis caused by heat stress mainly by reducing *MDM2*, thus affecting P53.

Then we used transcriptional profiling to find heat stress-induced pathways like defense response, response to external biotic stimulus, cytokine activity, regulation of immune system process, innate immune response, calcium ion binding, cell adhesion, and negative regulation of immune system process. The major enriched GO terms were involved in positive regulation of immune system process, immune system development, and hematopoietic or lymphoid organ development after RSV supplementation. It was suggested that the damage to broilers from chronic heat stress might be relieved by enhancing the immune system process, immune system development, and hematopoietic or lymphoid organ development. The results of the KEGG pathway enrichment analyses showed that the toll-like receptor signaling pathway and salmonella infection were key to RSV to respond to heat stress. Our previous study demonstrated that RSV could alleviate high-activated innate immunity and inflammatory response in yellow-feather broilers induced by heat stress via inhibiting the activation of splenic toll-like, C-type lectin-like, and NOD-like receptors signaling (6).

Innate immune PPRs-mediated signaling pathways (including NF-κB and MAPK pathways) are vital in inflammation responses under stressful conditions (5, 10). The present study showed that the RSV supplementation under heat stress significantly down-regulated the *NF-*κ*B, ERK, IKB-*α, *HSP70*, and *p38 MAPK* mRNA levels in the spleen compared with the HT group. LPS and RSV inhibited NF-κB activation and phosphorylation, as well as IKB*-*α degradation, and reduced nuclear content of NF-κB subunits in cells (60). In *in vitro* study, RSV reduced IL-1β levels and inhibited the NF-κB activation dependent on IL-1β (61, 62). Additionally, researches have shown that RSV may induce apoptosis and ROS accumulation through the p38 MAPK signaling pathway *in vitro* (63, 64). Under mastitis conditions, RSV was able to inhibit the inflammatory response by blocking the phosphorylation protein expression of p65 and IκB from the NF-κB signaling as well as phosphorylation of p38 MAPK and ERK from MAPK signaling (65). Our recent study also demonstrated that dietary RSV could inhibit NF-κB, MAPK, and PI3K/AKT signaling in broilers under heat stress conditions (6).

A higher level of HSP70 was reported in broilers under high-temperature stresses (5). HSP70 proteins can either activate the inflammatory immune system to fight infection or repress the immune system to prevent excessive inflammation (66, 67). Thus, HSPs were usually used as stress biomarkers to monitor cellular stress (12).

In particular, HSP70 plays a crucial role in preventing apoptotic progression induced by various environmental stresses (68). Apoptosome assembly is also a central step where HSPs can prevent apoptosome formation by selectively binding with Apaf-1 and by interacting with and inhibiting cytochrome c (69–71). Using a cell-free system, HSP70 inhibited cytochrome c/dATP-mediated caspase activation but allowed for the formation of Apaf-1 oligomers. HSP70 binds to Apaf-1 but not to procaspase-9, and suppresses recruitment of caspases to the apoptosome complex (69). Accordingly, the capacity of HSP70 to bind to specific proteins such as Apaf-1 raises its potential to inhibit caspase activation and suppress apoptosis (69). This interaction of apoptosis and cellular-stress responses probably stabilizes cell survival in response to injury.

## Conclusions

This study showed that heat stress harmed the growth performance and the immune capacity of the spleen in broilers. RSV supplementation had a beneficial effect on white feather broilers' growth performance and anti-inflammatory capacity, suggesting that it might effectively mitigate the deleterious effects of heat stress. RSV could inhibit heat stress-induced activation of splenic NF-κB, MAPK, and HSP70, as well as inhibit the activation of mitochondrial apoptotic pathways, thus reducing the splenic inflammatory response in heat-stressed white-feather broilers.

## Data Availability Statement

The datasets presented in this study can be found in online repositories. The names of the repository/repositories and accession number(s) can be found below: NCBI PRJNA804649.

## Ethics Statement

The animal study was reviewed and approved by Institutional Animal Care and Use Committee of the Hunan Agricultural University (permit number: CACAHU 2020-0821).

## Author Contributions

TM: conceptualization, methodology, formal analysis, data curation, and writing-original draft preparation. JD: sample collection and software. DX: visualization, investigation, and writing-review and editing. MA: writing-review and editing. CL and SH: conceptualization and methodology. LC and WD: methodology. JH: supervision, writing-reviewing and editing, project administration, and funding acquisition. All authors contributed to the article and approved the submitted version.

## Funding

This project was funded by the National Natural Science Foundation of China (31972600 and 31872991), National Natural Science Foundation of Hunan Province-China (2020JJ4364), Project of Science and Technology Department of Hunan Province (2020NK4247), and Double First Class Construction Project of Hunan Agricultural University (kxk201801004).

## Conflict of Interest

The authors declare that the research was conducted in the absence of any commercial or financial relationships that could be construed as a potential conflict of interest.

## Publisher's Note

All claims expressed in this article are solely those of the authors and do not necessarily represent those of their affiliated organizations, or those of the publisher, the editors and the reviewers. Any product that may be evaluated in this article, or claim that may be made by its manufacturer, is not guaranteed or endorsed by the publisher.
